# Association of Inadequately Low Left Ventricular Mass with Enhanced Myocardial Contractility in Severe Degenerative Aortic Stenosis

**DOI:** 10.3390/jcm7120464

**Published:** 2018-11-22

**Authors:** Bernadeta Chyrchel, Dorota Długosz, Klaudiusz Bolt, Olga Kruszelnicka, Artur Dziewierz, Jolanta Świerszcz, Ewa Wieczorek-Surdacka, Tomasz Hryniewiecki, Andrzej Surdacki

**Affiliations:** 1 Second Department of Cardiology, Jagiellonian University Medical College, 17 Kopernika Street, 31-501 Cracow, Poland; chyrchelb@gmail.com (B.C.); adziewierz@gmail.com (A.D.); 2 Students’ Scientific Group at the Second Department of Cardiology, Jagiellonian University Medical College, 17 Kopernika Street, 31-501 Cracow, Poland; dorotenka.1@gmail.com (D.D.); klaudiuszbolt@gmail.com (K.B.); 3 Department of Coronary Artery Disease and Heart Failure; The John Paul II Memorial Specialist Hospital, 80 Prądnicka Street, 31-202 Cracow, Poland; olga.kruszelnicka@onet.pl; 4 Department of Medical Education, Jagiellonian University Medical College, 16 Św. Łazarza Street, 31-530 Cracow, Poland; grasshoppers@interia.eu; 5 Chair and Department of Nephrology, Jagiellonian University Medical College, 15C Kopernika Street, 31-501 Cracow, Poland; esurdacka@gmail.com; 6 Department of Valvular Heart Defects, Institute of Cardiology,42 Alpejska Street, 04-628 Warsaw, Poland; thryniewiecki@ikard.pl

**Keywords:** aortic stenosis, echocardiography, left ventricular afterload, left ventricular hypertrophy, myocardial contractility

## Abstract

Background: Left ventricular hypertrophy (LVH), traditionally considered an adaptive mechanism that is aimed at the maintenance of LV systolic function, is absent in 10%–35% of patients with severe aortic stenosis (AS). Our aim was to estimate the clinical and hemodynamic characteristics in patients with severe AS and absent LVH, or inadequately low LV mass (i-lowLVM) relative to an individual hemodynamic load. Methods: We retrospectively analyzed in-hospital records of 100 patients with pure severe degenerative AS, preserved LV systolic function and without relevant coexistent diseases, except for well-controlled hypertension or diabetes. Results: Clinical characteristics were similar in patients with and without LVH, as well as those with and without i-lowLVM, except for slightly lower GFR at i-lowLVM. When compared to their counterparts, subjects without LVH or with i-lowLVM had smaller LV cavities, decreased LV wall thicknesses and higher EF. There were no significant differences in stenosis severity and indices of afterload (valvulo-arterial impedance and circumferential end-systolic LV wall stress), according to the presence or absence of either LVH or i-lowLVM. However, LV fractional shortening at the midwall level was elevated only in patients with i-lowLVM, but not in those without LVH, compared to the remainder (15.8 ± 3.3 vs. 12.9 ± 3.2%, *p* < 0.001 for those with and without i-lowLVM, respectively; 13.7 ± 3.7 vs. 13.8 ± 3.6% for LVH presence and absence, *p* = 0.9). Conclusions: Inadequately low LVM relative to the individual hemodynamic load could potentially reflect a different mode of the LV response to severe AS, associated with enhanced load-independent LV systolic performance, i.e., better LV contractility. If confirmed in a large series of patients, our small preliminary study may add to the possible mechanisms of a previously reported counterintuitive tendency of a lower, not higher, risk of adverse outcome in patients with low LV mass despite severe AS. Prospective studies are warranted, in order to determine a potential utility of LVM inadequacy in the risk stratification of patients with AS.

## 1. Introduction

Left ventricular hypertrophy (LVH), traditionally considered an adaptive mechanism that is aimed at the maintenance of LV systolic function, is absent in 10%−35% of patients with severe aortic stenosis (AS) [1,2,3,4], the second common cause of LV pressure overload. Additionally, a classical paradigm of a net benefit from LVH has been challenged by clinical and experimental data [5,6,7,8,9,10,11,12]. Importantly, the presence and magnitude of LVH is a well-recognized independent predictor of adverse cardiovascular (CV) outcome, including death and developing heart failure (HF), in population-based cohorts, hypertension, and AS [6,7,8,9,10,11,12]. This is compatible with reports of no excessive mortality [4], or even better early postoperative survival [2] and lower risk of adverse CV events [3] in patients with severe AS and absent LVH.

Moreover, patients with inadequately low LV mass (i-lowLVM), i.e., observed LV mass (LVM) in the lower part of the distribution of the value predicted from an individual hemodynamic load, had either a similar [13] or lower [3] risk of adverse CV events than their counterparts with an appropriate LVM in hypertension [13] and severe AS [3].

Notably, only a few studies have been focused on characteristics of patients with low LVM, despite severe AS [1,2,3,4]. Therefore, our aim was to estimate the clinical and hemodynamic characteristics in patients with severe AS and absent LVH or i-lowLVM.

## 2. Materials and Methods

### 2.1. Patients

We retrospectively analyzed in-hospital records of patients with pure severe degenerative AS (aortic valve area index [AVAI] <0.6 cm^2^/m^2^ or mean transvalvular pressure gradient >40 mmHg) without relevant coexistent diseases, except for well-controlled diabetes or hypertension, with preserved LV ejection fraction (≥50%) [14]. A complete list of exclusion criteria, including clinical or angiographic evidence of significant coronary artery disease (CAD) and estimated glomerular filtration rate (GFR) below 30 mL/min per 1.73 m^2^ (by the CKD-EPI formula), was reported earlier in detail [15]. Besides patients with severe AS out of the previously described AS subjects with moderate-to-severe AS [15], additional patients were also included into the final analysis (Bioethical Committee of Jagiellonian University, approval No. 122.6120.228.2016), provided that they fulfilled the inclusion and exclusion criteria.

### 2.2. Data Analysis

From routinely recorded parameters of LV structure and function, we calculated LV fractional shortening at the midwall level (mwFS), assuming a constant volume of the inner myocardial “shell” between the LV midwall and the endocardium, according to a simplified spherical LV model [16,17]. Additionally, estimated circumferential end-systolic LV wall stress (cESS) and valvulo-arterial impedance (Zva), indices of LV afterload, were computed. cESS was calculated from LV internal diameter and wall thickness at end-systole, averaged-in hospital systolic blood pressure (SBP), and maximal transvalvular aortic pressure gradient (APG_max_) [16,18,19,20], while Zva was derived from the SBP, mean transvalvular aortic pressure gradient, and stroke volume index [21]. Then, we computed a difference between the measured LVM (by the modified Devereux equation [22]) and that predicted from the height, gender, and an individual’s hemodynamic load (computed from a product of stroke volume and the sum of SBP and APG_max_) by a previously validated formula [20,23,24]. Accordingly, LVM_measured_ (g) = 0.8 × (1.04 × (((LVd + IVSd + PWd)^3^ − LVd^3^))) + 0.6, where LVd is the LV internal diameter, IVSd is the interventricular septum thickness, and PWSd is the posterior wall thickness (all measurements in end-diastole from the parasternal approach and expressed in cm) [22], while LV_predicted_ (g) = 55.37 + (0.009216 × (stroke volume (mL)) × (SBP + APG_max_ (mmHg))) + (6.63 × (height (m))^2.7^) − (18.1 − *n*), with *n* = 1 for men and *n* = 2 for women [3,17,20,23,24]. The difference between the measured and predicted LVM was termed an excess of LVM (eLVM), and expressed as a percentage of the predicted LVM, assumed to be 100%: eLVM = ((LVM_measured_ − LVM_predicted_)/LVM_predicted_) × 100% [17]. Because LVH, defined according to the classical mass criteria (i.e., LVM_measured_ >95 g/m^2^ for women and >115 g/m^2^ for men), was absent in 23% of our patients, for consistency, we assumed i-lowLVM as an eLVM below the 23th percentile of its distribution (i.e., eLVM <13%) in the study population. In addition to the indexation of LVM for body-surface area, we also repeated the analysis after the LVM normalization for height to the power of 2.7, as previously proposed [3,13,20].

### 2.3. Statistical Analysis

Data are shown as means and SD, or medians and interquartile range, and numbers with percentages. The analyzed subjects were compared according to the LVH presence or the LVM inadequacy by a 2-tailed Student’s *t*-test or the Mann–Whitney U-test, and Fisher’s test for continuous and categorical data, respectively. A Bonferroni-corrected *p*-value <0.05 was inferred significant, which corresponds to a pre-adjusted *p*-value <0.002.

## 3. Results

Clinical characteristics were similar in patients with and without LVH by the classical mass criteria, as well as with and without i-lowLVM relative to the individual hemodynamic load, except for slightly lower GFR in those with i-lowLVM (Table 1).

When compared to their counterparts, subjects without LVH or with i-lowLVM had smaller LV cavities, decreased LV wall thicknesses, and higher EF (Table 2). There were no significant differences in AVAI or in indices of afterload (cESS and Zva), according to the presence or absence of either LVH or i-lowLVM. However, mwFS was elevated only in patients with i-lowLVM, but not in those without LVH, in comparison to the remainder (Table 2).

The results were virtually unchanged when the LVH definition was based on the indexation of LVM for height instead of for body surface area.

## 4. Discussion

Our salient observation was an association of LVM inadequacy, but not LVH absence, with better LV systolic performance at the midwall level in severe AS. In addition, cESS and Zva—indices of afterload—were similar, while LV diastolic diameter, a raw measure of preload, was lower in AS patients with i-lowLVM than in their counterparts. Accordingly, this constellation of findings suggests that a load-independent increase in LV function, i.e., improved LV contractility, may be specific for AS subjects with i-lowLVM, and not for those with absent LVH.

Patients without LVH had smaller LV cavity sizes and thinner LV walls, as well as higher EF when compared to those with LVH, in agreement with previous reports [1,2,4]. Barasch et al. [4] speculated that some mechanisms could offset the detrimental effects of afterload excess in patients with severe AS and absent LVH, thereby explaining a tendency for better CV outcomes in that subset of AS subjects. Nevertheless, in our hands, cESS appeared to be unchanged in patients without LVH, which was secondary to LV concentric remodeling in the majority of this subgroup (as in earlier reports [1,2]), thus keeping cESS relatively constant. In addition, mwFS was also similar in patients with and without LVH. This indicates that, despite the absence of LVH, LV afterload and myocardial systolic function are preserved, while a higher EF in AS patients free of LVH seems rather to be a consequence of concentric LV geometry and better LV chamber function, not improved myocardial performance.

In contrast, patients with i-lowLVM exhibited increased both EF and mwFS in comparison to their counterparts, with a ratio of measured to predicted LVM above the 23th percentile of its distribution in the study population. This appears to be partially analogous to an early report by De Simone et al. [13] who described a higher cESS-corrected mwFS in hypertensive patients with low LVM, defined as an eLVM below −32% (corresponding to the 2.5th percentile of eLVM distribution in a reference population) despite similar LV end-diastolic diameter, a raw index of preload. That observation [13] was indicative of better LV contractility, as in our patients with i-lowLVM, who exhibited increased mwFS, unchanged cESS, and even smaller LV diameter, compared to the remainder. Of note, in those hypertensive subjects with low LVM, mean RWT was as low as 0.28 [13], while in our AS subjects with i-lowLVM, RWT averaged 0.47, reflecting concentric LV remodeling in most of them. As cESS was not elevated in AS patients with i-lowLVM, it may be concluded that—unlike in hypertension—concentric remodeling appears to be necessary to preserve normal cESS in the majority of patients with severe AS and i-lowLVM. With regard to a potential mechanism of this observation, it can be speculated that severe AS results in a fixed and probably more potent LV pressure overload, in contrast to hypertension, frequently associated with fluctuating blood pressure.

Notably, excessive LVH, i.e., inappropriately high LVM out of proportion to LV afterload, is associated with depressed mwFS, despite a lower or unchanged cESS, indicating depressed LV myocardial performance in patients with mild-to-severe AS [3,19,20], which has also recently been confirmed by our group in moderate AS [17]. Therefore, our results could reflect a continuum of the relationship between LV contractility, wall stress, and LVH adequacy, in agreement with a concept that was previously proposed by Aurigemma et al. [18,25], De Simone et al. [13], and Palmieri et al. [26] for hypertension, and later by Mureddu et al. [19] and Cioffi et al. [3,20] for AS. On the basis of LV stress-shortening relations, they suggested that excessive LVH may be a compensatory, albeit largely ineffective compensatory mechanism triggered by primary myocardial dysfunction, and aimed at restoring LV systolic performance through lowering LV wall stress. Consequently, this mechanism would not be operational in subjects with enhanced LV contractility, thereby contributing to the association of apparently low LVM with increased cESS-corrected mwFS in patients with hypertension [13]. Hence, our report is the first to demonstrate a similar mechanism in AS, another common cause of LV pressure overload.

The enhanced LV contractile function might be protective against LV dysfunction in severe AS with i-lowLVM. In accordance with this hypothesis, Kupari et al. [2] observed an increased EF, a 3-fold lower prevalence of HF, and a better 3-month postoperative outcome in patients with a lack of LVH, despite critical AS. Additionally, in an early report, Seiler and Jenni [1] described a better ergometric working capacity in AS patients with severe AS and no LVH. Unfortunately, the cited authors [1,2] estimated only the LVH presence, and not the LVM adequacy, which limits more detailed comparisons with the present study. Nonetheless, apart from this limitation, the proposed hypothesis can explain a lower risk of adverse CV events (whose majority consisted of aortic valve replacement and HF hospitalizations) in patients with severe AS in the lower tertile of the measured-to-predicted LVM ratio (<108%, i.e., eLVM <8%) [3], close to the cut-off value for i-lowLVM assumed in the present study (eLVM <13%). Consequently, LVM “inadequacy” can represent not a lack of putatively “compensatory” LVH, but rather, a different mode of LV response to pressure overload, as proposed by Kupari et al. [2] on the basis of an absence of LVM regression after aortic valve replacement in patients without LVH.

Due to a paucity of studies, determinants of absent LVH and especially i-lowLVM still remain unexplored. In agreement with earlier observations [1,2], the percentage of men in AS subjects without LVH was insignificantly higher than in those with LVH. A similar, albeit weaker, tendency was observed in AS patients with i-lowLVM. To the best of our knowledge, sex-dependent differences in the prevalence of i-lowLVM in severe AS have not been reported so far [3]. However, as a higher proportion of men had been described in AS with inappropriately high LVM versus appropriate LVM [3,20], it may be suggested that the adequacy of the LV hypertrophic response to chronic LV overload in AS appears to be modulated by gender.

### Limitations

First, a retrospective design and a relatively low number of patients are major limitations of our report. However, exclusively subjects without coexistent diseases, including CAD, were entered into the final analysis, in order to reduce the heterogeneity of the study group. Second, the individual hemodynamic load was estimated on the basis of in-hospital BP recordings and echocardiography, while averaged values over a long period of time would be more appropriate. Nevertheless, we analyzed only patients with well-controlled hypertension and stable BP, which suggests that the computed parameters could be, to some degree, representative for a given subject. Third, medical therapy, which is known to affect LVM, was not uniform. However, the proportions of patients treated with renin–angiotensin system inhibitors, drugs with a well-recognized ability to attenuate LVH, were similar, according to LVH presence or LVM inadequacy.

## 5. Conclusions

Keeping in mind the limitations of our small retrospective study, inadequately low LVM relative to the individual hemodynamic load could potentially reflect a different mode of LV adaptation to AS, associated with enhanced load-independent LV systolic performance, i.e., better LV contractility. If confirmed in a large series of patients, our preliminary report might add to the possible mechanisms of a previously reported counterintuitive tendency of a lower, not higher, risk of adverse outcome in patients with low LV mass, despite severe AS. Prospective studies are warranted in order to determine a potential utility of LVM inadequacy for the prediction of clinical outcomes and risk stratification of patients with AS.

## Figures and Tables

**Table 1:** Demographic and clinical characteristics according to LVH absence or LVM inadequacy.

Characteristic	LV Hypertrophy (LVH)			Inadequately Low LVM (i-lowLVM)		
	No *n* = 23	Yes *n* = 77	*p*	Yes *n* = 23	No *n* = 77	*p*
Age, years	69 ± 8	70 ± 10	NS	72 ± 6	69 ± 11	NS
Men/women, n (%)	14 (61%)	34 (44%)	NS	13 (56%)	35 (45%)	NS
BSA, m^2^	1.9 ± 0.1	1.8 ± 0.2	NS	1.9 ± 0.1	1.8 ± 0.2	NS
BMI, kg/m^2^	30.4 ± 4.4	29.0 ± 4.6	NS	29.3 ± 4.4	29.5 ± 4.6	NS
Hypertension, n (%)	20 (87%)	64 (83%)	NS	21 (91%)	63 (82%)	NS
Diabetes, n (%)	7 (30%)	22 (29%)	NS	9 (39%)	20 (26%)	NS
GFR, mL/min/1.73 m^2^	70 ± 16	77 ± 19	0.09	67 ± 15	78 ± 20	0.02
Symptoms, n (%)	14 (61%)	37 (48%)	NS	15 (65%)	36 (47%)	NS
Mean BP, mm Hg	95 ± 11	92 ± 9	NS	94 ± 11	92 ± 9	NS
Medications, n (%)						
ACEI or ARB	7 (30%)	20 (26%)	NS	9 (39%)	18 (23%)	NS
Beta-blocker	13 (57%)	44 (57%)	NS	15 (65%)	42 (55%)	NS
Diuretics	12 (52%)	36 (47%)	NS	15 (65%)	33 (43%)	0.07

Data are shown as mean ± standard deviation or numbers (percentages). ACEI: angiotensin-converting enzyme inhibitor; ARB: angiotensin receptor blocker; BMI: body mass index; BP: blood pressure; BSA: body surface area; GFR: estimated glomerular filtration rate; *n*: number; NS: non-significant.

**Table 2:** Echocardiographic characteristics and LV afterload according to the presence of LVH or LVM inadequacy.

Characteristic	LV Hypertrophy (LVH)			Inadequately Low LVM (i-lowLVM)		
	No *n* = 23	Yes *n* = 77	*p*	Yes *n* = 23	No *n* = 77	*p*
AVAI, cm^2^/m^2^	0.5 ± 0.1	0.4 ± 0.1	NS	0.5 ± 0.1	0.4 ± 0.1	NS
LVd, cm	4.4 ± 0.4	5.0 ± 0.7	**<0.001**	4.6 ± 0.6	5.0 ± 0.8	0.03
PWd, cm	1.1 ± 0.1	1.3 ± 0.2	**<0.001**	1.0 ± 0.1	1.3 ± 0.2	**<0.001**
IVSd, cm	1.1 ± 0.2	1.5 ± 0.3	**<0.001**	1.1 ± 0.2	1.5 ± 0.3	**<0.001**
RWT	0.50 ± 0.07	0.57 ± 0.16	0.07	0.47 ± 0.06	0.58 ± 0.15	**0.001**
EF, %	63 ± 11	58 ± 7	0.004	64 ± 10	57 ± 7	**<0.001**
mwFS, %	13.8 ± 3.6	13.7 ± 3.7	NS	15.8 ± 3.3	12.9 ± 3.2	**<0.001**
cESS, hPa	201 ± 81	178 ± 92	NS	188 ± 72	181 ± 97	NS
Zva, mmHg / mL/m^2^	6.0 ± 1.5	5.4 ± 1.9	NS	5.5 ± 1.2	5.4 ± 2.0	NS
eLVM, %	8 [−2,24]	56 [26,81]	**<0.001**	5 [−6,9]	57 [29,81]	**<0.001**

Data are shown as mean ± standard deviation or median ± interquartile range. Significant Bonferroni-corrected *p*-values are marked in bold. AVAI: aortic valve area index; cESS: circumferential end-systolic LV wall stress; EF: ejection fraction; eLVM: excess of LV mass; IVSd: interventricular septum thickness at end-diastole; LV: left ventricular; LVd: LV end-diastolic diameter; LVM: LV mass; mwFS: LV midwall fractional shortening; *n*: number; NS: non-significant; PWd: posterior wall thickness at end-diastole; RWT: relative LV wall thickness; Zva: valvulo-arterial impedance.
